# Machine learning model for predicting immediate postoperative desaturation using spirometry signal data

**DOI:** 10.1038/s41598-023-49062-9

**Published:** 2023-12-11

**Authors:** Youmin Shin, Yoon Jung Kim, Juseong Jin, Seung-Bo Lee, Hee-Soo Kim, Young-Gon Kim

**Affiliations:** 1 Department of Transdisciplinary Medicine, Seoul National University Hospital, 101 Daehak-Ro, Jongno-Gu, Seoul, 03080 Republic of Korea; 2 Interdisciplinary Program in Bio-engineering, Seoul National University, Seoul, Republic of Korea; 3 Department of Anesthesiology and Pain Medicine, Seoul National University Hospital, College of Medicine, Seoul National University, 101 Daehak-Ro, Jongno-Gu, Seoul, 03080 Republic of Korea; 4 Integrated Major in Innovative Medical Science, Seoul National University, Seoul, Republic of Korea; 5 Department of Medical Informatics, Keimyung University School of Medicine, Daegu, Republic of Korea; 6 Department of Medicine, Seoul National University College of Medicine, Seoul, Republic of Korea

**Keywords:** Computational biology and bioinformatics, Health care, Medical research

## Abstract

Postoperative desaturation is a common post-surgery pulmonary complication. The real-time prediction of postoperative desaturation can become a preventive measure, and real-time changes in spirometry data can provide valuable information on respiratory mechanics. However, there is a lack of related research, specifically on using spirometry signals as inputs to machine learning (ML) models. We developed an ML model and postoperative desaturation prediction index (DPI) by analyzing intraoperative spirometry signals in patients undergoing laparoscopic surgery. We analyzed spirometry data from patients who underwent laparoscopic, robot-assisted gynecologic, or urologic surgery, identifying postoperative desaturation as a peripheral arterial oxygen saturation level below 95%, despite facial oxygen mask usage. We fitted the ML model on two separate datasets collected during different periods. (Datasets A and B). Dataset A (Normal 133, Desaturation 74) was used for the entire experimental process, including ML model fitting, statistical analysis, and DPI determination. Dataset B (Normal 20, Desaturation 4) was only used for verify the ML model and DPI. Four feature categories—signal property, inter-/intra-position correlation, peak value/interval variability, and demographics—were incorporated into the ML models via filter and wrapper feature selection methods. In experiments, the ML model achieved an adequate predictive capacity for postoperative desaturation, and the performance of the DPI was unbiased.

## Introduction

Laparoscopic and robot-assisted approaches are commonly used in lower abdominal surgery^1,2^. These procedures require pneumoperitoneum and a steep Trendelenburg position^3^. An increase in intra-abdominal pressure and head-down position causes displacement of the diaphragm to the cephalad, promotes atelectasis, and reduces lung compliance and arterial oxygenation^4–6^. Pneumoperitoneum can decrease dynamic lung compliance and functional residual capacity^7,8^. When the functional residual capacity falls below the closing capacity, there is an increased risk of small airway obstruction and atelectasis^4^.

A previous prospective study reported that postoperative desaturation occurred in 37% of the patients undergoing laparoscopic surgery^9^. Perioperative desaturation is associated with myocardial infarction and acute kidney injury^10,11^. Postoperative desaturation is a common pulmonary complication after surgery and may persist because of poor patient outcomes, such as brain damage and increased mortality^12–15^.

Modern anesthesia delivery systems provide real-time spirometry monitoring^16^. The spirometry signals exhibit changes owing to pneumoperitoneum and the steep Trendelenburg position. Specifically, the peak inspiratory pressure (PIP) significantly increases after position changes^7,8^; thus, patient positions can be classified based on the PIP signal. While real-time changes in spirometry data can offer valuable insights into respiratory mechanics, it's important to note that research utilizing spirometry signals has, until recently, remained exceptionally rare in the field.

A feasible early or real-time prediction of postoperative desaturation can become a preventive measure through appropriate anesthetic management interventions. Additionally, using spirometry signals as inputs for machine learning (ML) models has not been extensively studied. We anticipate contributing not only to improving patient prognoses through postoperative desaturation prediction but also to interpreting the significance of spirometry signals and expanding their clinical relevance.

In this study, we aimed to develop an ML model using intraoperative spirometry signal data, including PIP, airway pressure (AWP), and lung volume (VOL), for the Supine and Trendelenburg positions to predict the risk of immediate postoperative desaturation. We considered accessibility and clinical usefulness to select these signals. PIP, AWP, and VOL are easily accessible data during mechanical ventilation. PIP is manifested as one value per cycle per breath quantitatively, whereas AWP is collected not only during inspiration but also during expiration. Although PIP can be derived from AWP, we assumed that using PIP in conjunction with AWP would be useful to utilize the information. AWP and volume form a spirometry loop, providing information regarding lung mechanics. In addition, previous studies reported that mechanical power was associated with ventilator-related lung injury^17–19^. AWP and VOL both play an important role in calculating mechanical power^20^.

We applied various feature extraction methods to utilize signal information as inputs to the model and selected important features through feature selection. Furthermore, we aimed to derive an index from the selected features for use in clinical settings, referred to as the postoperative desaturation prediction index (DPI).

The remainder of this paper is organized as follows. Section "Results" outlines the material and methods. Sections "Discussion" and "Conclusions" present the results and discussions, respectively. Section "Materials and methods" concludes the study.

## Results

During the study period from January 2020 to April 2022, we analyzed 207 cases (Supplementary Fig. 1). Patient characteristics are summarized in Table 1. Postoperative desaturation in the post-anesthesia care unit occurred in 74 of the 207 patients.

**Table 1:** Patient characteristics.

Characteristic	(N = 207)
Age, yr (range)	63 (20–84)
Weight, kg	64.7 (10.2)
Height, cm	163 (7.1)
BMI, kg/cm2	24.3 (3.2)
Sex, female	92 (44.4%)
Operation	
Gynecologic surgery	89 (43.0%)
Urologic surgery	117 (56.5%)
General surgery	1 (0.5%)
Surgical modality	
Robot-approach	118 (57.0%)
Laparoscopic approach	89 (43.0%)
Underlying disease	
Hypertension	66 (31.9%)
Diabetes mellitus	33 (15.9%)
Liver disease	15 (7.2%)
Lung disease	2 (1%)
Asthma	1 (0.5%)
Heart disease	6 (2.9%)
Thyroid disease	35 (16.9%)
Renal disease	6 (2.9%)
Neurologic disease	4 (1.9%)
Obesity	82 (39.6%)
Anemia	6 (2.9%)
ASA-PS classification	
1	39 (18.8%)
2	159 (76.8%)
3	9 (4.3%)
Anesthesia time, h	3.0 [2.3–3.7]
Operation time, h	2.3 [1.8–3.0]
Urine output, mL	224 (207)
Estimated blood loss, mL	314 (242)
Infused volume, mL	1205 (670)

Data are expressed as number (percentage), mean (standard deviation), or median (interquartile range).

*ASA-PS* American Society of Anesthesiologists physical status classification system.

**Figure 1:**
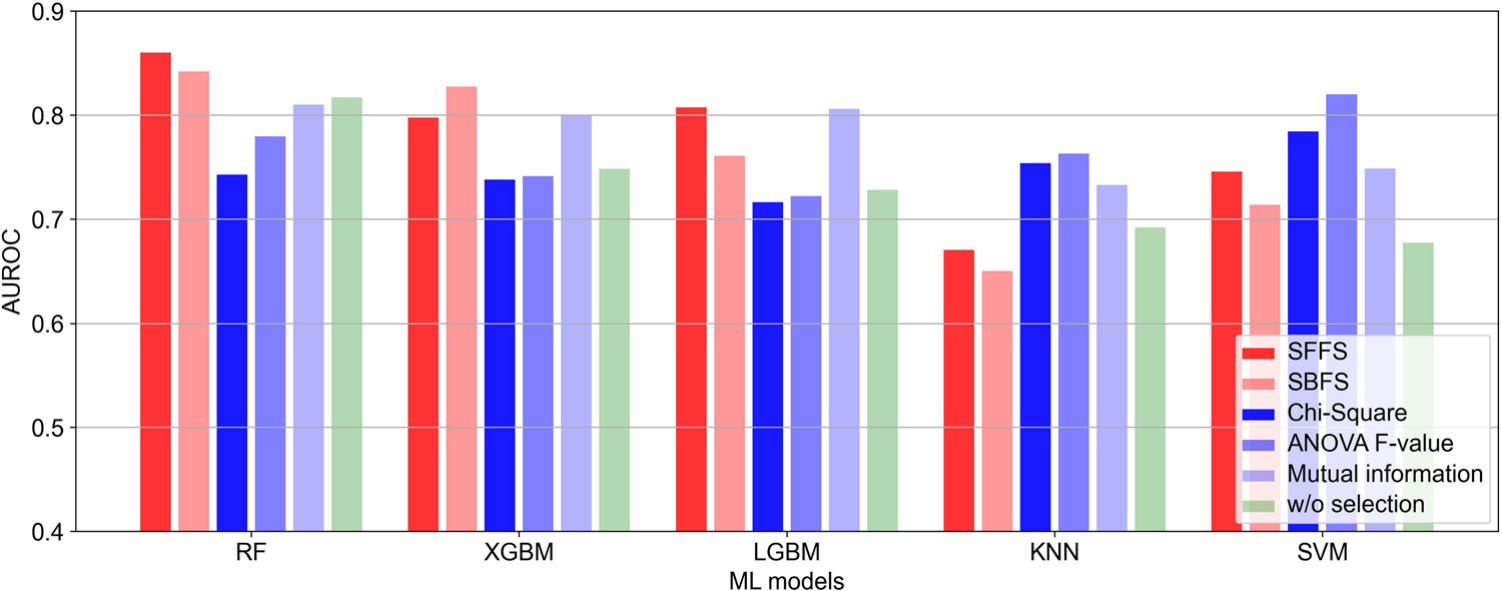
Performance comparison in terms of AUROC. The figure shows the results of feature selection using wrapper [represented in red: sequential forward feature selection (SFFS), sequential backward feature selection (SBFN)], and filter methods (represented in blue, Chi-square, ANOVA F-value, Mutual information) and the results of without any selection (represented in green).

Figure 1 shows the classification performance of the five models with respect to the different feature selection methods. When the sequential forward floating selection (SFFS) method, a wrapper-based feature selection method, was applied to the random forest (RF) model, the highest performance (AUROC = 0.856 (95% confidence interval: 0.764–0.948)) was obtained. The classification performance on Dataset B was AUROC = 0.812 (95% confidence interval: 0.740–0.885).

Ten features were selected including three from PIP, four from VOL, two from AWP, and one representing the relationship between VOL and AWP. Based on the patient position, seven were included for the Supine position, two for the Trendelenburg position, and one for the inter-position correlation (Table 2). Table 3 summarizes the results of the statistical analysis between the training and test sets of Dataset A, and normal and postoperative desaturation patients for the test set. None of the 10 selected features exhibited significant differences between the training and test set distributions. Out of the 10 features, six were statistically significant (*p*-value < 0.05). An additional LASSO feature selection found that five of the 10 features selected above were erased. Consequently, the five selected features are the outlier occurrence rate (Z-score based) of the PIP signal acquired during the Supine segment, the mean value of the PIP signal acquired during the Supine section, the median value of the volume signal acquired during the Supine section, the peak interval variability (PIV) of the volume signal acquired during the Supine section, and the mean value of the AWP signal acquired during the Trendelenburg section.

**Table 2:** Selected feature list with the highest performance in RF model and SFFS selection.

Feature index	Position	Signal	Feature type	Feature name
F1	Supine	PIP	Signal property	Z-score based Outlier ratio
F2	Supine	PIP	Signal property	Mean
F3	Supine	VOL	Signal property	Median
F4	Supine	VOL	Variability	PVV (standard deviation)
F5	Supine	VOL	Variability	PIV (root mean square of successive differences)
F6	Supine	VOL, AWP	Intra-position Correlation	KC
F7	Supine	AWP	Variability	Peak value variability (standard deviation)
F8	Trendelenburg	PIP	Signal property	Standard deviation
F9	Trendelenburg	AWP	Signal property	Mean
F10	Supine, Trendelenburg	VOL	Inter-position Correlation	PCC

*PIP* peak inspiratory pressure, *VOL* lung volume, *AWP* airway pressure, *PVV* peak value variability, *PIV* peak interval variability, *KC* Kendall rank correlation coefficient, *PCC* Pearson correlation coefficient.

**Table 3:** Statistical analysis for selected feature shown in Table 1 (“v” indicates features selected using LASSO feature selection).

Feature index	Train	Test	*p* value	Normal	PD	*p* value	LASSO
F1	1681 [782 to 3428]	1773 [703 to 4307]	0.386	1601 [606 to 3439]	2105 [795 to 4581]	0.119	v
F2	14.8 [13.8 to 15.9]	15.3 [14.1 to 16.4]	0.067	14.7 [13.9 to 15.9]	15.7 [14.8 to 16.4]	< 0.001	v
F3	121.6 (37.2)	126.4 (32.2)	0.409	119.3 (32.7)	136.4 (31.8)	< 0.001	v
F4	65.4 [13.1 to 102.0]	64.7 [11.8 to 108.4]	0.466	47.2 [9.8 to 98.7]	99.5 [45.0 to 130.7]	< 0.001	
F5	22.3 [12.5 to 44.0]	29.3 [16.0 to 63.0]	0.058	24.3 [13.6 to 56.8]	36.1 [16.2 to 66.6]	0.428	v
F6	0.52 (0.06)	0.53 (0.06)	0.119	0.53 (0.06)	0.54 (0.07)	0.256	
F7	1.41 [1.18 to 2.56]	1.38 [0.86 to 1.98]	0.071	1.29 [0.83 to 2.05]	1.47 [1.13 to 2.00]	0.029	
F8	2.03 [1.56 to 3.12]	2.20 [1.80 to 2.84]	0.370	2.16 [1.69 to 2.89]	2.18 [1.82 to 2.77]	0.356	
F9	11.5 [10.3 to 14.2]	11.5 [10.3 to 12.8]	0.316	10.8 [9.9 to 12.1]	12.6 [11.5 to 14.3]	< 0.001	v
F10	0.00 [− 0.04 to 0.01]	− 0.00 [− 0.02 to 0.02]	0.268	0.00 [− 0.01 to 0.02]	− 0.00 [− 0.10 to 0.00]	< 0.001	

Data are expressed as numbers (percentages), means (standard deviations), or medians (interquartile ranges).

*PD* postoperative desaturation.

The remaining five features were used for DPI suggestion. DPI can be calculated using Eq. (1) when the regression coefficients of ${\text{C}}_{{{\text{Outlier\,ratio\,of\,PIP}}}}^{{{\text{Supine}}}} ,{\text{C}}_{{{\text{Mean\,of\,PIP}}}}^{{{\text{Supine}}}} ,{\text{C}}_{{{\text{Median\,of\,VOL}}}}^{{{\text{Supine}}}} ,{\text{C}}_{{{\text{PIV\,of\,VOL}}}}^{{{\text{Supine}}}}$, and ${\text{C}}_{{{\text{Mean\,of\,AWP}}}}^{{{\text{Trendelenburg}}}}$ are 8.00e-06, 3.79e-02, respectively, where F1, F2, F3, F5 and F9 denote the values of each feature (Supplementary Table 1):

1 $$ {\text{DPI}} = {\text{C}}_{{{\text{Outlier\,ratio \,PIP}}}}^{{{\text{Supine}}}} *F1 + {\text{C}}_{{{\text{Mean\,of\,PIP}}}}^{{{\text{Supine}}}} *F2 + {\text{C}}_{{{\text{Median\,of\,VOL}}}}^{{{\text{Supine}}}} *F3 + {\text{C}}_{{{\text{PIV\,of\,VOL}}}}^{{{\text{Supine}}}} *F5 + {\text{C}}_{{{\text{Mean\,of\,AWP}}}}^{{{\text{Trendelenburg}}}} *F9 $$

**Figure 2:**
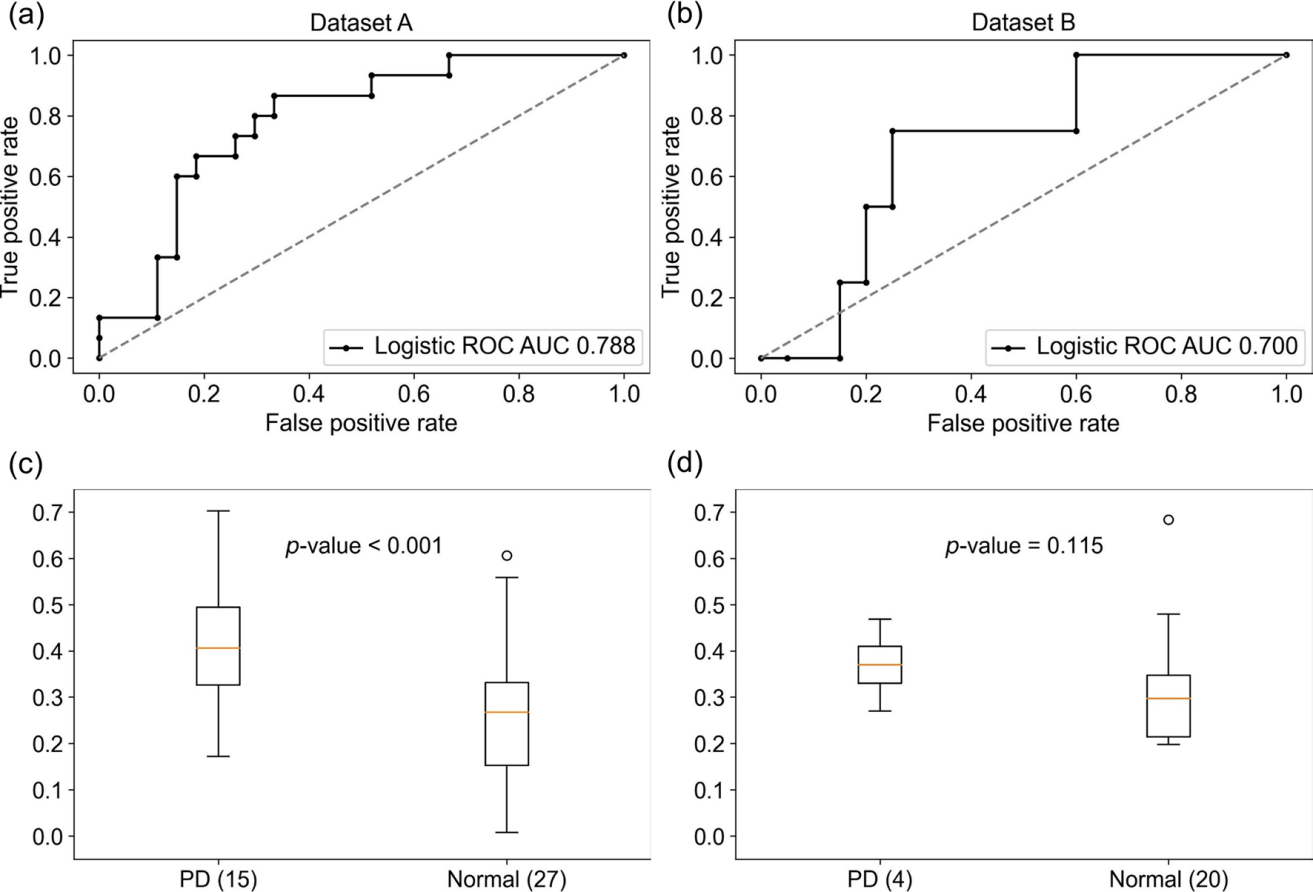
Performance comparison of DPI. (**a**, **c**) DPI performance on the test set of Dataset A (**a** AUROC, **c** statistic). (**b**, **d**) DPI performance on Dataset B (**b** AUROC, **d** statistic).

The clinical applicability of DPI using the selected features is shown in Fig. 2. In terms of prediction, DPI achieved AUROCs of 0.788 (95% confidence interval: 0.734–0.841) and 0.700 (95% confidence interval: 0.650–0.750) on the test sets of Datasets A and B, respectively. The statistical significance of DPI performance was confirmed with a *p*-value of 0.001 for Dataset A, but no significance was shown with a *p*-value of 0.115 for Dataset B. The diagnostic performance of DPI for Dataset A was the highest when the threshold was set to 0.4, yielding an accuracy of 0.762, sensitivity of 0.600, specificity of 0.852, and PPV of 0.692. Supplementary Table 2 provides detailed information on the diagnostic performance across different threshold adjustments.

## Discussion

We developed an ML model and DPI to predict postoperative desaturation in patients who underwent robotic or laparoscopy-assisted lower abdominal surgeries using intraoperative spirometry signals. This study represents a novel approach to assessing the predictive capability of intraoperative spirometry for postoperative desaturation.

We attempted to extract the maximum number of features and subsequently employed a feature selection process to identify the most significant features. In the absence of feature selection, the RF model demonstrated superior performance (AUROC = 0.817). As shown in Fig. 1, when selecting features using the filter method, the support vector machine (SVM) model achieved the highest performance with an MI reference feature selection (AUROC = 0.819). The filter method can reduce the dimensionality of datasets^21^, and is simple and fast (Supplementary Table 3)^22^.

The RF model achieved the best performance (AUROC = 0.856 and accuracy (ACC) = 0.810) when SFFS, a wrapper-based feature selection method, was applied. Although wrapper feature selection is computationally expensive and prone to overfitting, it typically outperforms the filter method^21,23,24^.

As summarized in Table 3, approximately half of the selected features exhibited no statistically significant differences between the normal and postoperative desaturation groups. Although the filter method considers only the properties of individual features, wrapper feature selection is based on the performance achieved by attempting several combinations of features. The filter method can prevent overfitting effectively. However, it often fails to select the best features, as it overlooks feature interactions, assumes linear relationships, is insensitive to the learning algorithm, and lacks iterative feedback. Consequently, only a wrapper-based method can select features that are not statistically significant. Therefore, the wrapper method generally outperforms the filter method^24^.

In our experiment, RF outperformed the other ML models. Although their performances can vary depending on the dataset, RF outperformed extreme gradient boosting model (XGBM) and light gradient boosting model (LGBM) for several reasons: RF performs better when the features are highly correlated, has a built-in mechanism to handle correlated features effectively, and tends to be less sensitive to model complexity and hyperparameter setting. Furthermore, RF can handle high-dimensional data and reduce overfitting, whereas XGBM and LGBM may struggle with collinearity among features^25,26^. Among the 10 features selected through wrapper-based feature selection, five were filtered from the additional LASSO selection to obtain the DPI. In situations of high-collinearity, RF can prevent overfitting and achieve superior performance. XGBM and LGBM performed poorly on the test set than on the training set, and RF performed similarly on both sets (Supplementary Fig. 2).

In terms of prediction, DPI achieved an AUROC of 0.788 on the test set of Dataset A, exhibiting a statistically significant difference between normal and postoperative desaturation patients (*p*-value = 0.001); however, the performance slightly degraded on Dataset B (AUROC = 0.700, *p*-value = 0.115). Owing to a lack of sufficient samples, specifically only four patients with postoperative desaturation, conducting a meaningful performance comparison presented a significant challenge. We believed that presenting a feasible index before the occurrence of an adverse event is important. For usability purposes, index that is quantifiable with consistency is desirable; therefore, we offered the DPI. In addition, this study could be a basis for further research to investigate whether DPI can improve patient outcomes.

The main features selected were the outlier ratio and average value of the PIP signal acquired from the Supine position, rather than during the pneumoperitoneum and head-down position. The initiation of pneumoperitoneum and the head-down position rapidly decreased lung compliance^7,8^. Here, the absolute increase in PIP caused by pneumoperitoneum and the head-down position could potentially pose challenges for ML algorithms in distinguishing between desaturation and non-desaturation cases.

Our study revealed an intriguing finding: the variability in the respiration cycle while in the Supine position was chosen as a feature in our model. Volume signal variability is represented by the peak interval of the volume curve, which corresponds to the duration of each breath. Our analysis focused solely on the mechanical ventilation periods. This finding might suggest that modulation of the respiratory rate before the surgical procedure affects the pulmonary system. High respiratory rates are reported to be associated with reduced ventilation-induced lung injuries^27^, and respiratory rate is also known to be an important factor in the calculation of mechanical power, a concept that has recently gained attention^17,28^. However, the impact of its variability is still unknown, and further research is necessary. Although the ML approach employed in our study could not distinguish the specific cause, the respiratory rate may have changed to eliminate carbon dioxide or correct hyperventilation. To further evaluate the effects of changes in respiratory rate on pulmonary mechanics, a prospective study with better control over other variables is warranted.

Another feature selected is the median volume value. Extending the time to maintain a high volume could be unfavorable for lung protection. High tidal volume affects respiratory system compliance and is associated with increased mortality and fewer ventilator-free days^29,30^. Recently, there has been a growing argument that the titration volume is more crucial than PEEP titration for lung protection, which aligns with the findings of our model^31^. Valuable information on factors associated with postoperative desaturation can be extracted from PIP and VOL signals in the Supine position obtained in the early intraoperative period. The capacity to partially explain postoperative desaturation using pre-existing features offers optimism for the feasibility of early interventions, which could potentially lead to improved patient outcomes.

Among the parameters obtained during pneumoperitoneum and the head-down position, the AWP signal was selected. Changes in position and intra-abdominal pressure led to a sudden focal increase in AWP. This increase in AWP may be attributed to diminished compliance and resistance^32^. Excessive AWP can potentially result in barotrauma, which provides a rationale for selecting it as a feature. Moreover, most mechanical ventilation machines incorporate pressure limits to mitigate the risk of barotrauma^33^. This limit reduces the tidal volume when excessive AWP is detected, and such insufficient ventilation could subsequently lead to the development of atelectasis. Consequently, patients exhibiting significant fluctuations in AWP may be more susceptible to postoperative desaturation.

This study has several limitations. First, the interpretations given to the acquired DPI and selected features need to be validated. As there are no relevant prior studies to draw upon for interpreting the results of this study, the conclusions can only be tentative. Further clinical studies are required to confirm these findings. Second, we did not verify whether the same results could be achieved using other institutions or other equipment. We evaluated the performance of the ML model and DPI for another holdout dataset (Dataset B), and all data were collected from different periods with the same equipment. Although external validation from other institutions would have been ideal, we encountered challenges in finding an institution that allowed retrospective review of volume and AWP data through the VitalDB repository with consistent ventilator settings. A prospective study is necessary to validate these results.

## Conclusions

This study demonstrated the potential of spirometry signals in improving patient prognosis by predicting postoperative desaturation. These signals hold significance in terms of interpreting their meaning and expanding their importance. Our ML model exhibited fair predictive ability on Datasets A and B; however, the performance of DPI was not satisfactory on Dataset B owing to a significant data imbalance, posing challenges for accurate analysis. To validate our model and DPI, further large-scale studies are essential.

## Materials and methods

### Data sources

This retrospective study was approved (number: 2206-090-1332) by the Institutional Review Board (IRB) of Seoul National University Hospital, Seoul, Korea on June 16, 2022. The patient data was anonymized prior to analysis, and the IRB of the Seoul National University Hospital waived the requirement for informed consent. Our study followed the principles of the Declaration of Helsinki and adhered to the Strengthening the Reporting of Observational Studies in Epidemiology (STROBE) Statement.

### Datasets

The study cohort comprised patients who underwent laparoscopic, robot-assisted gynecologic, or urologic surgery at Seoul National University Hospital from January 2020 to April 2022. The inclusion criteria were patients undergoing laparoscopic or robot-assisted surgery. The exclusion criteria were as follows: missing data of oxygen saturation via pulse oximetry in the post-anesthesia care unit; short surgery duration (< 1.5 h); surgery converted into the open method; patients with unexpected events during surgery, such as excessive bleeding and intraoperative lung injury; and technical error in collecting spirometry data. Oxygen desaturation in the post-anesthesia care unit was defined as SpO_2_ < 95% despite facial oxygen mask application^34,35^. Details of patient inclusion and exclusion are provided in Supplementary Fig. 1.

Two datasets acquired from the same institution at different time points were used.

1. *Dataset A*: This was used for the entire experimental process, including ML model fitting, statistical analysis, and DPI suggestion. Out of 207 patient observations, 42 (Normal 27, Desaturation 15) randomly extracted patient observations were designated as the test set, and the remaining 165 (Normal 106, Desaturation 59) data samples were divided using five-fold stratification to select features and evaluate ML models.
2. *Dataset B*: This was used to verify the experimental results. We collected data from 24 patients (Normal 20, Desaturation 4) at the same institution during different periods.

Data are expressed as numbers (percentages), means (standard deviations), or medians (interquartile ranges). The term ASA-PS refers to the American Society of Anesthesiologists physical status classification system.

### Data preprocessing

During surgery, bio-signals were acquired with VitalDB^36^. The spirometry signals were converted into digital form at a sampling frequency of 15 Hz. The position separation rules were as follows:

1. *Supine position section start*: The initial time point when the PIP value reaches or exceeds 0.3 and the difference between successive PIP values remains at 0.1 or less for 5 min
2. *Supine position section end*: If the PIP value exceeds 0.7 consecutively after the start of the Supine position section
3. *Trendelenburg position section start*: The starting point of a high PIP value, when the PIP value is ≥ 0.7 for 5 min
4. *Trendelenburg position section end*: After the start of the Trendelenburg position, when two consecutive drops in the PIP values are less than 0.5

To reduce the errors that may occur in position separation owing to the fluctuation of the PIP signal, we applied Gaussian smoothing ($\upsigma = 60 \times {\text{sampling\,frequency}}$) to decrease noise and variations in the dataset. The smoothed PIP signal was normalized using minimum–maximum scaling. Signal extraction at the correct position was important for the analysis. To reflect spirometry signal changes due to position alterations and to improve the accuracy of the rule base, only 80% of the center of each “Supine” was considered.

### Feature extraction

**Figure 3:**
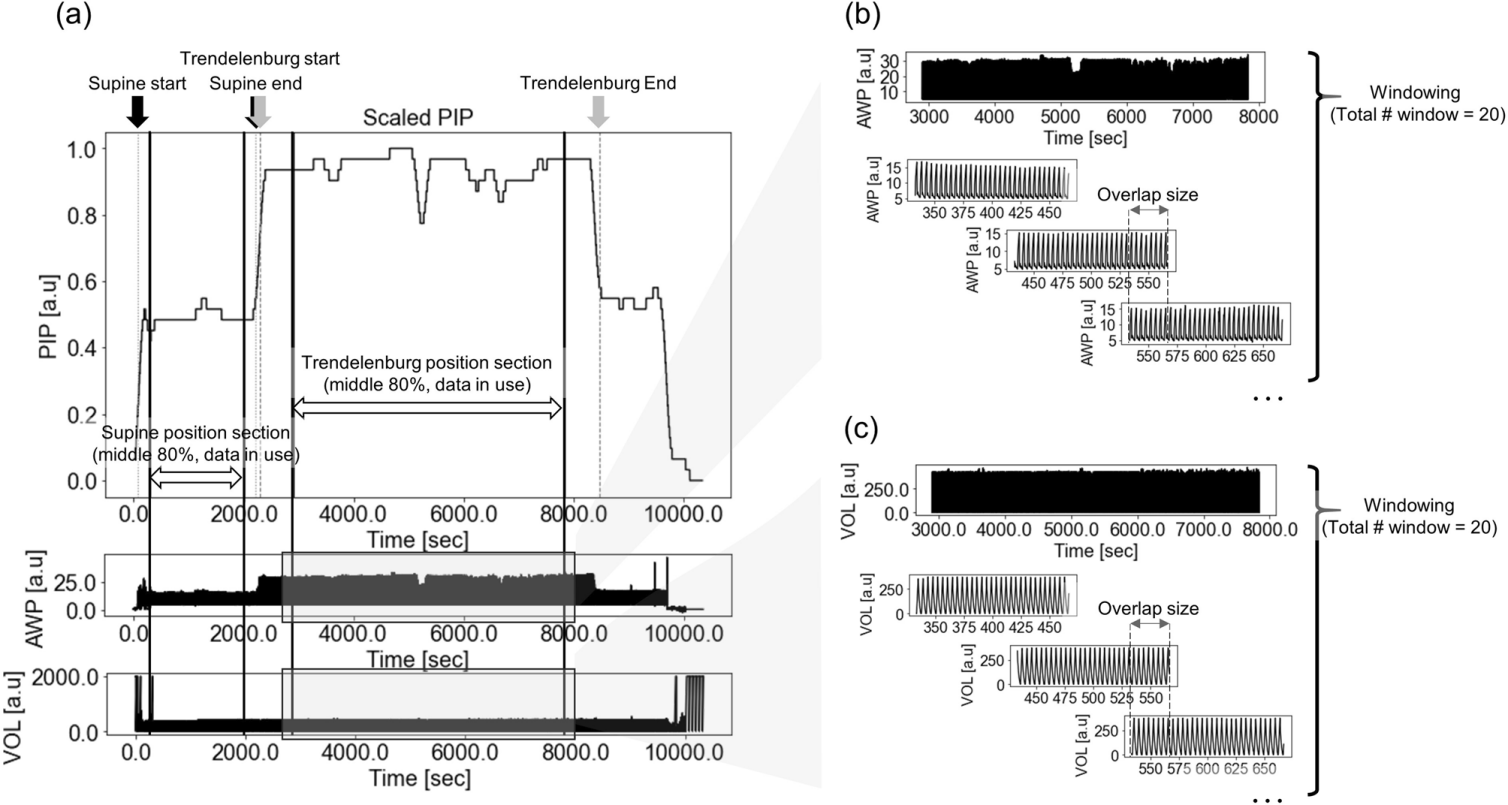
Data preprocessing and feature extraction diagram. (**a**) Supine and Trendelenburg position sections are separated based on the PIP signal, and 80% of the center of each position section was used to reflect spirometry signal changes and to improve the inaccuracy of the rule base. (**b**, **c**) Each Supine and Trendelenburg position section were divided into 20 windows.

To adjust for the difference in the length of individual surgical time and obtain stable features, we divided the entire Supine and Trendelenburg position sections into 20 windows. Each window comprised 2000 observations ($\approx 133.3\,{\text{s}}, 2000/{\text{sampling\,frequency}}$). The window-to-window overlap size was adjusted based on the length of each position (${\text{size}} = \frac{{{\text{length}}\;{\text{of}}\;{\text{position}} - {\text{window}}\;{\text{size}}}}{{{\text{total}}\# \;{\text{window}}}}$). For samples larger than 400,000, we maximized the distance between each window. Except for demographic features and the total signal length, the average value from the extracted 20 windows was used as the input for the ML models (Fig. 3b,c).

The features we extracted in this study were largely divided into four types: Detailed information is presented in Supplementary Table 4.

1. *Demographic features*: A total of four features regarding the clinical information of patients such as sex, age, weight, and height were used.
2. *Signal property*: A total of 44 features regarding signal properties such as total length, average, median, skewness, kurtosis, standard deviation, and outlier ratios of the signals were calculated.
3. *Demographic features*: A total of four features regarding the clinical information of patients such as sex, age, weight, and height were used.
4. *Correlations*: A total of 20 features regarding the positional correlation were extracted.
  Inter-position correlations: The correlations for the windows obtained in the Supine and Trendelenburg positions were calculated. The Pearson correlation coefficient (PCC), Spearman correlation coefficient (SCC), Kendall rank correlation coefficient (KC), coherence, and dynamic time warping (DTW) were calculated.
  Intra-position correlation: This correlation was obtained in the Supine and Trendelenburg positions. A correlation analysis between AWP and VOL was performed using PCC, SCC, KC, coherence, and DTW.
5. *Peak value/interval variability*: A total of 108 features were extracted regarding peak-related variabilities. Peak detection was performed for each AWP and VOL signal to extract the peak values and their intervals. Variability features were extracted for both peak values and intervals using the heart rate variability analysis function, the measure of the variation in time intervals between consecutive heartbeats using both time- and frequency domain measures^37^.

Feature extraction was conducted solely on the signal properties of PIP signals. They are not periodic signals, and the correlation between the PIP, AWP, and VOL signals has no clinical significance.

### Feature selection

Feature selection was performed to improve the performance of the ML model and reduce the risk of overfitting. A total of 176 features were used, and feature selection was performed using two approaches (Supplementary Fig. 3) commonly used in classification tasks:

1. *Filter methods*: Filtering was performed without a predictive model^24^. Three commonly used strategies (i.e., Chi-Square, ANOVA F-value, and mutual information) were employed.
2. *Wrapper methods*: Wrapper methods use a search algorithm to evaluate different subsets of features and select the optimal subset that achieves the best performance for a given ML model. SFFS and sequential backward floating selection, which generally exhibit high performance, were adopted^23,38^.

### Classification

Five different commonly used ML models in the healthcare field were used, namely RF, XGBM, LGBM,* k*-nearest neighbors (KNN), and SVM^39,40^. The setting of the hyperparameters was focused on preventing overfitting. Detailed information on hyperparameters is presented in Supplementary Table 5. The optimal feature selection method and resulting selected features were designated based on the average area under the receiver operating characteristic curve (AUROC) during five-fold stratification.

### Statistical analysis

Based on a normality test using the Shapiro–Wilk test, an independent *T*-test and Mann–Whitney statistical analysis were performed for normal and non-normal distribution features, respectively. For a small sample size, the normality test may more likely reject the null hypothesis. To address this, we set a stringent significance level (*p*-value = 0.01) for the Shapiro–Wilk test.

### DPI suggestion

The DPI, which provides only quadratic operations of selected features, can be used as a more intuitive and reliable prediction tool. After LASSO feature selection^41^, the DPI was presented through multivariate linear regression and fitted based on the mean squared error to minimize the residual sum of squares between observed and predicted targets^42^. LASSO selection is an embedded feature selection method that is more suitable for regression than classification because it removes the multicollinearity between features^43^.

### Supplementary Information

Supplementary Information.

## Data Availability

The datasets used and/or analyzed during the current study available from the corresponding author on reasonable request.
