# Supplementary material for: Machine learning model for predicting immediate postoperative desaturation using spirometry signal data
Source: Sci Rep. 2023 Dec 11;13:21881. doi: 10.1038/s41598-023-49062-9 (PMC10711018; doi:10.1038/s41598-023-49062-9)
Supplement: Supplementary file 1 — Supplementary Information. [file 41598_2023_49062_MOESM1_ESM.docx]

Supplementary materials


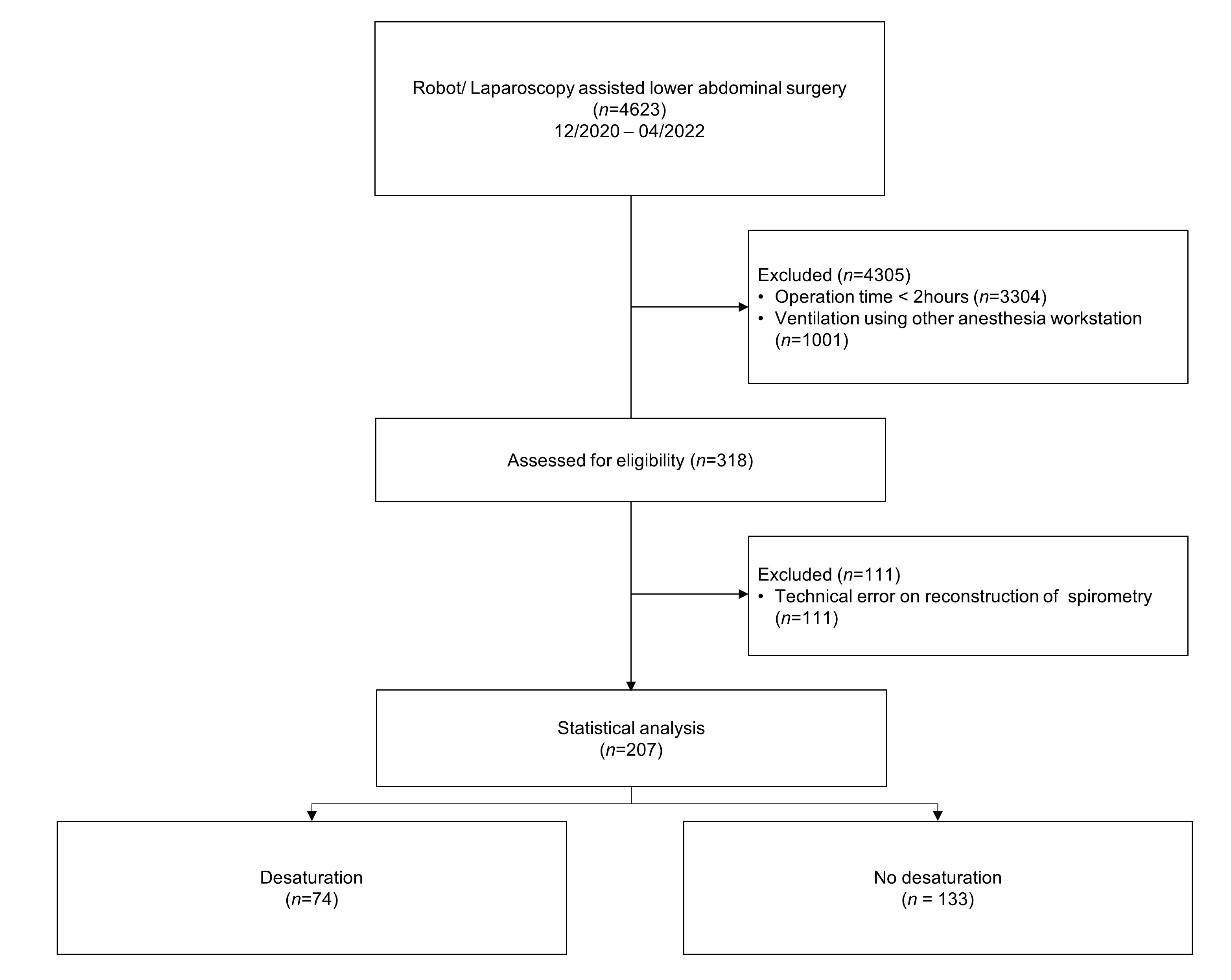


**Supplementary Figure 1. Patient inclusion and exclusion flow**


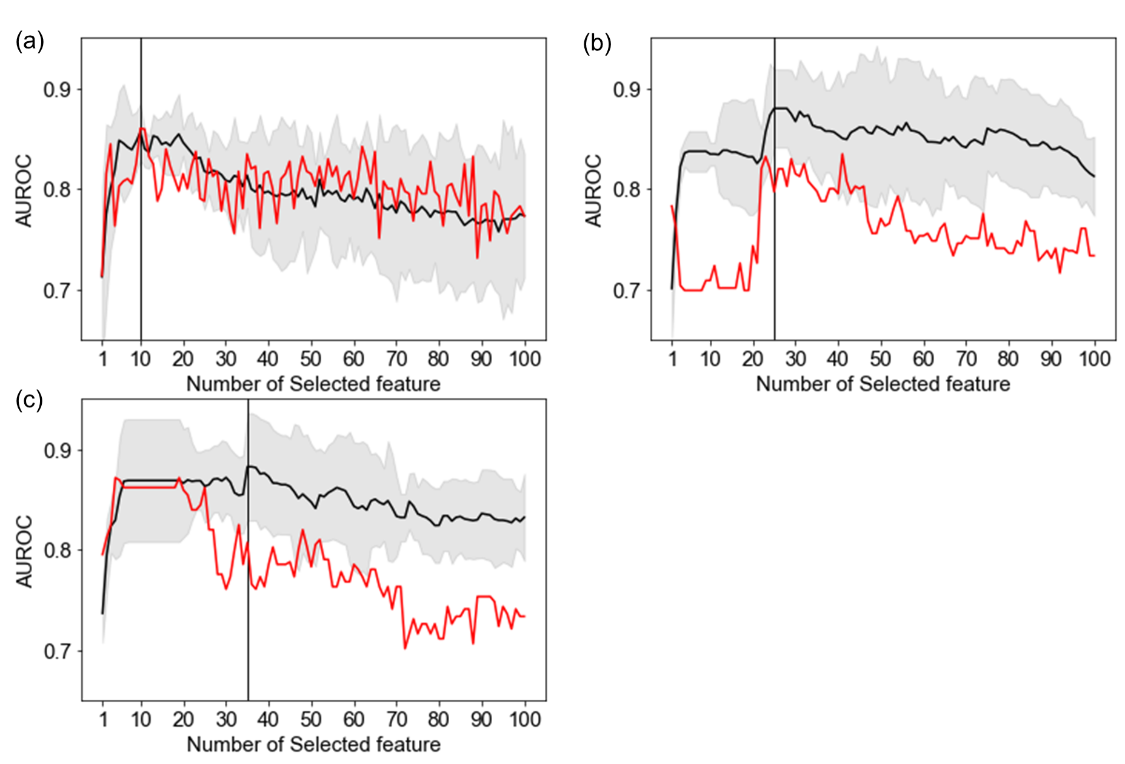


**Supplementary Figure 2. Classification performance using the wrapper method for feature selection.** The black line represents the mean and standard deviation obtained during the 5-fold training, while the red line indicates performance on the test set. (a) Random forest, (b) XGBM, and (c) LGBM.


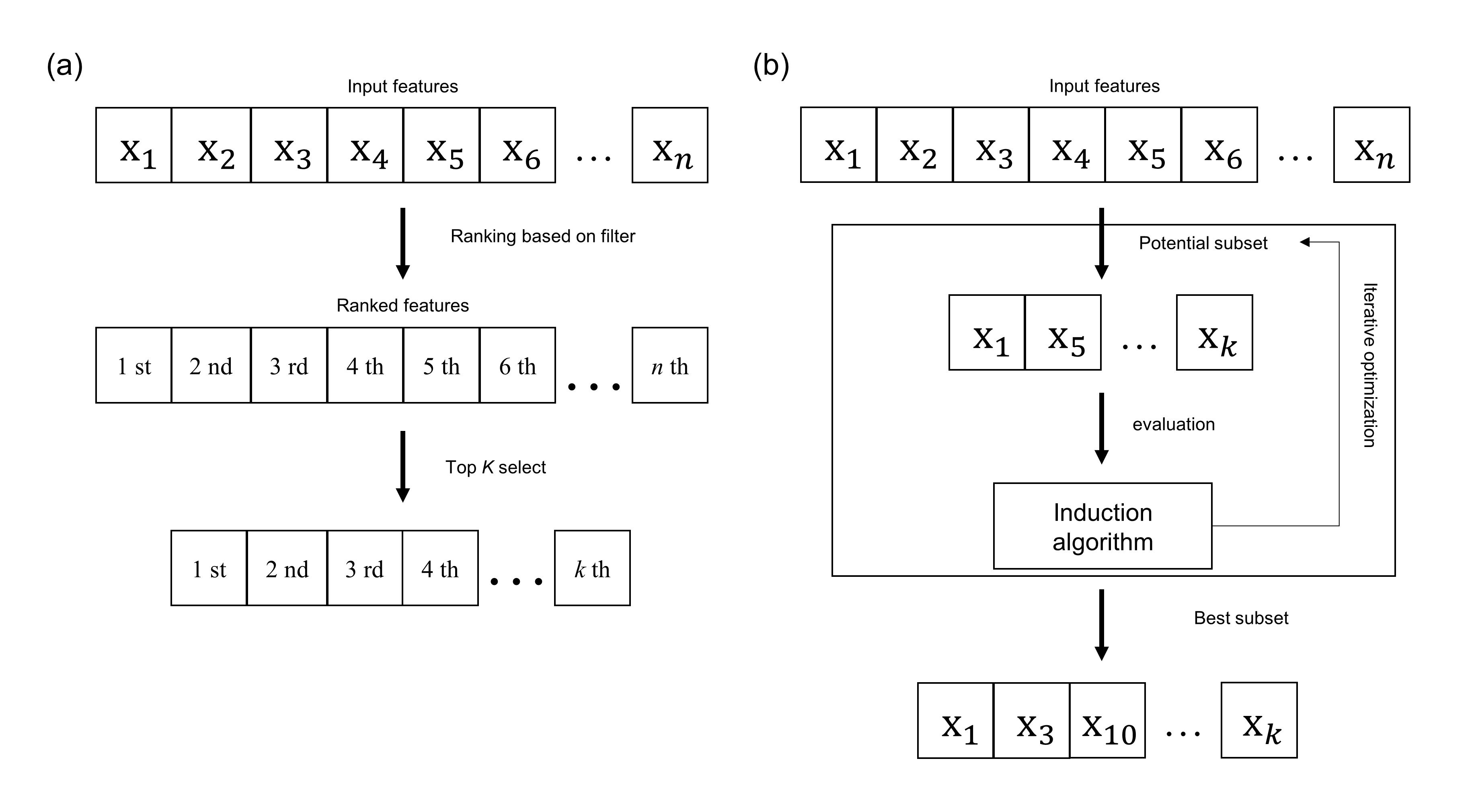


**Supplementary Figure 3. Schematic diagram of feature selection.** (**a**) filter-based feature selection, (**b**) wrapper-based feature selection

Supplementary Table 1. DPI calculation formula and coefficients of each feature

| Feature value | Coefficients |
| --- | --- |
| F1 | $\mathbf{C}_{\mathbf{Outlier ratio of PIP}}^{\mathbf{Supine}}$: 8.00e-06 |
| F2 | $\mathbf{C}_{\mathbf{Mean of PIP}}^{\mathbf{Supine}}$: 3.79e-02 |
| F3 | $\mathbf{C}_{\mathbf{Median of VOL}}^{\mathbf{Supine}}$: 2.30e-03 |
| F5 | $\mathbf{C}_{\mathbf{PIV of VOL}}^{\mathbf{Supine}}$: 2.43e-03 |
| F9 | $\mathbf{C}_{\mathbf{Mean of AWP}}^{\mathbf{Trendelenburg}}$: 1.44e-02 |
| $\mathbf{DPI=}\mathbf{C}_{\mathbf{Outlier ratio of PIP}}^{\mathbf{Supine}}\mathbf{*F1 +}\mathbf{C}_{\mathbf{Mean of PIP}}^{\mathbf{Supine}}\mathbf{*F2+}\mathbf{C}_{\mathbf{Mean of VOL}}^{\mathbf{Supine}}\mathbf{*F3+}\mathbf{C}_{\mathbf{PIV of VOL}}^{\mathbf{Supine}}\mathbf{*F4+}\mathbf{C}_{\mathbf{Mean of AWP}}^{\mathbf{Trendelenburg}}\mathbf{*F5}$ | |

DPI: postoperative desaturation prediction index, PIP: peak inspiratory pressure, VOL: lung volume, PIV: peak interval variability, AWP: airway pressure.

Supplementary Table 2. DPI performance across different threshold adjustment

| Threshold | AUROC | Accuracy | Sensitivity | Specificity | PPV | NPV |
| --- | --- | --- | --- | --- | --- | --- |
| 0.100 | 0.788 | 0.452 | 1.000 | 0.148 | 0.395 | 1.000 |
| 0.200 |  | 0.571 | 0.933 | 0.370 | 0.452 | 0.909 |
| 0.300 |  | 0.714 | 0.867 | 0.630 | 0.565 | 0.895 |
| 0.400 |  | 0.762 | 0.600 | 0.852 | 0.692 | 0.793 |
| 0.500 |  | 0.667 | 0.267 | 0.889 | 0.571 | 0.686 |

DPI: postoperative desaturation prediction index, AUROC: area under the receiver operating characteristic curve, PPV: positive predictive value, NPV: negative predictive value.

**Supplementary Table 3. Comparison of the speed of feature selection methods**

| Model | Feature selection methods | | Running time [sec] |
| --- | --- | --- | --- |
| RF | Filter method | Chi-Square | 42.386 |
|  | Filter method | ANOVA F-value | 41.266 |
|  | Filter method | Mutual information | 106.683 |
|  | Wrapper | SFFS | 3404.904 |
|  | Wrapper | SBFS | 3494.048 |
| XGBM | Filter method | Chi-Square | 39.748 |
|  | Filter method | ANOVA F-value | 33.269 |
|  | Filter method | Mutual information | 94.354 |
|  | Wrapper | SFFS | 6517.162 |
|  | Wrapper | SBFS | 1900.230 |
| LGBM | Filter method | Chi-Square | 18.767 |
|  | Filter method | ANOVA F-value | 15.702 |
|  | Filter method | Mutual information | 93.551 |
|  | Wrapper | SFFS | 1858.412 |
|  | Wrapper | SBFS | 1120.398 |
| KNN | Filter method | Chi-Square | 6.761 |
|  | Filter method | ANOVA F-value | 6.984 |
|  | Filter method | Mutual information | 72.380 |
|  | Wrapper | SFFS | 232.271 |
|  | Wrapper | SBFS | 101.930 |
| SVM | Filter method | Chi-Square | 5.659 |
|  | Filter method | ANOVA F-value | 4.941 |
|  | Filter method | Mutual information | 71.000 |
|  | Wrapper | SFFS | 188.454 |
|  | Wrapper | SBFS | 139.013 |

RF: random forest, XGBM: extreme gradient boosting model, LGB: light gradient boosting model, KNN: *K*-nearest neighbors, SVM: support vector machine, SFFS: sequential forward floating selection, SBFS: sequential backward floating selection.

Supplementary Table 4. Total features in use

| Index | Category | Name | Description |
| --- | --- | --- | --- |
| 1 | Demographic | age |  |
| 2 | Demographic | sex |  |
| 3 | Demographic | height |  |
| 4 | Demographic | weight |  |
| 5 | Signal property | pip_len_sup | Total length of Supine PIP |
| 6 | Signal property | pip_len_tren | Total length of Trendelenburg PIP |
| 7 | Signal property | pip_sup_iqr_ratio | Outlier (IQR based) ratio during Supine PIP |
| 8 | Signal property | pip_tren_iqr_ratio | Outlier (IQR based) ratio during Trendelenburg PIP |
| 9 | Signal property | pip_sup_Z_ratio | Outlier (Z-score based) ratio during Supine PIP |
| 10 | Signal property | pip_tren_Z_ratio | Outlier (Z-score based) ratio during Trendelenburg PIP |
| 11 | Signal property | pip_sup_skew | Average skewness of Supine PIP |
| 12 | Signal property | pip_tren_skew | Average skewness of Trendelenburg PIP |
| 13 | Signal property | pip_sup_kurto | Average kurtosis of Supine PIP |
| 14 | Signal property | pip_tren_kurto | Average kurtosis of Trendelenburg PIP |
| 15 | Signal property | pip_sup_mean | Average mean of Supine PIP |
| 16 | Signal property | pip_tren_mean | Average mean of Trendelenburg PIP |
| 17 | Signal property | pip_sup_median | Average median of Supine PIP |
| 18 | Signal property | pip_tren_median | Average median of Trendelenburg PIP |
| 19 | Signal property | pip_sup_sd | Average standard deviation of Supine PIP |
| 20 | Signal property | pip_tren_sd | Average standard deviation of Trendelenburg PIP |
| 21 | Signal property | vol_sup_iqr_ratio | Outlier (IQR based) ratio during Supine VOL |
| 22 | Signal property | vol_tren_iqr_ratio | Outlier (IQR based) ratio during Trendelenburg VOL |
| 23 | Signal property | vol_sup_Z_ratio | Outlier (Z-score based) ratio during Supine VOL |
| 24 | Signal property | vol_tren_Z_ratio | Outlier (Z-score based) ratio during Trendelenburg VOL |
| 25 | Signal property | vol_sup_skew | Average skewness of Supine VOL |
| 26 | Signal property | vol_tren_skew | Average skewness of Trendelenburg VOL |
| 27 | Signal property | vol_sup_kurto | Average kurtosis of Supine VOL |
| 28 | Signal property | vol_tren_kurto | Average kurtosis of Trendelenburg VOL |
| 29 | Signal property | vol_sup_mean | Average mean of Supine VOL |
| 30 | Signal property | vol_tren_mean | Average mean of Trendelenburg VOL |
| 31 | Signal property | vol_sup_median | Average median of Supine VOL |
| 32 | Signal property | vol_tren_median | Average median of Trendelenburg VOL |
| 33 | Signal property | vol_sup_sd | Average standard deviation of Supine VOL |
| 34 | Signal property | vol_tren_sd | Average standard deviation of Trendelenburg VOL |
| 35 | Signal property | awp_sup_iqr_ratio | Outlier (IQR based) ratio during Supine AWP |
| 36 | Signal property | awp_tren_iqr_ratio | Outlier (IQR based) ratio during Trendelenburg AWP |
| 37 | Signal property | awp_sup_Z_ratio | Outlier (Z-score based) ratio during Supine AWP |
| 38 | Signal property | awp_tren_Z_ratio | Outlier (Z-score based) ratio during Trendelenburg AWP |
| 39 | Signal property | awp_sup_skew | Average skewness of Supine AWP |
| 40 | Signal property | awp_tren_skew | Average skewness of Trendelenburg AWP |
| 41 | Signal property | awp_sup_kurto | Average kurtosis of Supine AWP |
| 42 | Signal property | awp_tren_kurto | Average kurtosis of Trendelenburg AWP |
| 43 | Signal property | awp_sup_mean | Average mean of Supine AWP |
| 44 | Signal property | awp_tren_mean | Average mean of Trendelenburg AWP |
| 45 | Signal property | awp_sup_median | Average median of Supine AWP |
| 46 | Signal property | awp_tren_median | Average median of Trendelenburg AWP |
| 47 | Signal property | awp_sup_sd | Average standard deviation of Supine AWP |
| 48 | Signal property | awp_tren_sd | Average standard deviation of Trendelenburg AWP |
| 49 | Correlation | intra_sup_pearson | Pearson correlation between Supine section AWP and VOL |
| 50 | Correlation | intra_tren_pearson | Pearson correlation between Trendelenburg section AWP and VOL |
| 51 | Correlation | inter_vol_pearson | Pearson correlation between Supine VOL and Trendelenburg VOL |
| 52 | Correlation | inter_awp_pearson | Pearson correlation between Supine AWP and Trendelenburg AWP |
| 53 | Correlation | intra_sup_tau | Tau correlation between Supine section AWP and VOL |
| 54 | Correlation | intra_tren_tau | Tau correlation between Trendelenburg section AWP and VOL |
| 55 | Correlation | inter_vol_tau | Tau correlation between Supine VOL and Trendelenburg VOL |
| 56 | Correlation | inter_awp_tau | Tau correlation between Supine AWP and Trendelenburg AWP |
| 57 | Correlation | intra_sup_spear | Spearman correlation between Supine section AWP and VOL |
| 58 | Correlation | intra_tren_spear | Spearman correlation between Trendelenburg section AWP and VOL |
| 59 | Correlation | inter_vol_spear | Spearman correlation between Supine VOL and Trendelenburg VOL |
| 60 | Correlation | inter_awp_spear | Spearman correlation between Supine AWP and Trendelenburg AWP |
| 61 | Correlation | intra_sup_coh | Coherence between Supine section AWP and VOL |
| 62 | Correlation | intra_tren_coh | Coherence between Trendelenburg section AWP and VOL |
| 63 | Correlation | inter_vol_coh | Coherence between Supine VOL and Trendelenburg VOL |
| 64 | Correlation | inter_awp_coh | Coherence between Supine AWP and Trendelenburg AWP |
| 65 | Correlation | intra_sup_dtw | DTW between Supine section AWP and VOL |
| 66 | Correlation | intra_tren_dtw | DTW between Trendelenburg section AWP and VOL |
| 67 | Correlation | inter_vol_dtw | DTW between Supine VOL and Trendelenburg VOL |
| 68 | Correlation | inter_awp_dtw | DTW between Supine AWP and Trendelenburg AWP |
| 69 | Variability | pv_sup_awp_0 | Variability features (Time domain - 'mean nni') in peak values of Supine AWP |
| 70 | Variability | pv_sup_awp_1 | Variability features (Time domain - 'rmssd') in peak values of Supine AWP |
| 71 | Variability | pv_sup_awp_2 | Variability features (Time domain - 'median nni') in peak values of Supine AWP |
| 72 | Variability | pv_sup_awp_3 | Variability features (Time domain - 'mean hr') in peak values of Supine AWP |
| 73 | Variability | pv_sup_awp_4 | Variability features (Time domain - 'max hr') in peak values of Supine AWP |
| 74 | Variability | pv_sup_awp_5 | Variability features (Time domain - 'min hr') in peak values of Supine AWP |
| 75 | Variability | pv_sup_awp_6 | Variability features (Time domain - 'std hr') in peak values of Supine AWP |
| 76 | Variability | pv_sup_awp_7 | Variability features (Time domain - 'total number of peak') in peak values of Supine AWP |
| 77 | Variability | pv_sup_awp_8 | Variability features (Time domain - 'outlier (IQR based) ratio of peak') in peak values of Supine AWP |
| 78 | Variability | pv_sup_awp_9 | Variability features (Time domain - 'outlier (Z-score based) ratio of peak') in peak values of Supine AWP |
| 79 | Variability | pv_sup_awp_10 | Variability features (Frequency domain - 'lf') in peak values of Supine AWP |
| 80 | Variability | pv_sup_awp_11 | Variability features (Frequency domain - 'hf') in peak values of Supine AWP |
| 81 | Variability | pv_sup_awp_12 | Variability features (Frequency domain - 'lf hf ratio') in peak values of Supine AWP |
| 82 | Variability | pv_sup_awp_13 | Variability features (Frequency domain - 'total power') in peak values of Supine AWP |
| 83 | Variability | pi_sup_awp_0 | Variability features (Time domain - 'mean nni') in peak intervals of Supine AWP |
| 84 | Variability | pi_sup_awp_1 | Variability features (Time domain - 'rmssd') in peak intervals of Supine AWP |
| 85 | Variability | pi_sup_awp_2 | Variability features (Time domain - 'median nni') in peak intervals of Supine AWP |
| 86 | Variability | pi_sup_awp_3 | Variability features (Time domain - 'mean hr') in peak intervals of Supine AWP |
| 87 | Variability | pi_sup_awp_4 | Variability features (Time domain - 'max hr') in peak intervals of Supine AWP |
| 88 | Variability | pi_sup_awp_5 | Variability features (Time domain - 'min hr') in peak intervals of Supine AWP |
| 89 | Variability | pi_sup_awp_6 | Variability features (Time domain - 'std hr') in peak intervals of Supine AWP |
| 90 | Variability | pi_sup_awp_7 | Variability features (Time domain - 'outlier (IQR based) ratio of peak') in peak intervals of Supine AWP |
| 91 | Variability | pi_sup_awp_8 | Variability features (Time domain - 'outlier (Z-score based) ratio of peak') in peak intervals of Supine AWP |
| 92 | Variability | pi_sup_awp_9 | Variability features (Frequency domain - 'lf') in peak intervals of Supine AWP |
| 93 | Variability | pi_sup_awp_10 | Variability features (Frequency domain - 'hf') in peak intervals of Supine AWP |
| 94 | Variability | pi_sup_awp_11 | Variability features (Frequency domain - 'lf hf ratio') in peak intervals of Supine AWP |
| 95 | Variability | pi_sup_awp_12 | Variability features (Frequency domain - 'total power') in peak intervals of Supine AWP |
| 96 | Variability | pv_sup_vol_0 | Variability features (Time domain - 'mean nni') in peak values of Supine VOL |
| 97 | Variability | pv_sup_vol_1 | Variability features (Time domain - 'rmssd') in peak values of Supine VOL |
| 98 | Variability | pv_sup_vol_2 | Variability features (Time domain - 'median nni') in peak values of Supine VOL |
| 99 | Variability | pv_sup_vol_3 | Variability features (Time domain - 'mean hr') in peak values of Supine VOL |
| 100 | Variability | pv_sup_vol_4 | Variability features (Time domain - 'max hr') in peak values of Supine VOL |
| 101 | Variability | pv_sup_vol_5 | Variability features (Time domain - 'min hr') in peak values of Supine VOL |
| 102 | Variability | pv_sup_vol_6 | Variability features (Time domain - 'std hr') in peak values of Supine VOL |
| 103 | Variability | pv_sup_vol_7 | Variability features (Time domain - 'total number of peak') in peak values of Supine VOL |
| 104 | Variability | pv_sup_vol_8 | Variability features (Time domain - 'outlier (IQR based) ratio of peak') in peak values of Supine VOL |
| 105 | Variability | pv_sup_vol_9 | Variability features (Time domain - 'outlier (Z-score based) ratio of peak') in peak values of Supine VOL |
| 106 | Variability | pv_sup_vol_10 | Variability features (Frequency domain - 'lf') in peak values of Supine VOL |
| 107 | Variability | pv_sup_vol_11 | Variability features (Frequency domain - 'hf') in peak values of Supine VOL |
| 108 | Variability | pv_sup_vol_12 | Variability features (Frequency domain - 'lf hf ratio') in peak values of Supine VOL |
| 109 | Variability | pv_sup_vol_13 | Variability features (Frequency domain - 'total power') in peak values of Supine VOL |
| 110 | Variability | pi_sup_vol_0 | Variability features (Time domain - 'mean nni') in peak intervals of Supine VOL |
| 111 | Variability | pi_sup_vol_1 | Variability features (Time domain - 'rmssd') in peak intervals of Supine VOL |
| 112 | Variability | pi_sup_vol_2 | Variability features (Time domain - 'median nni') in peak intervals of Supine VOL |
| 113 | Variability | pi_sup_vol_3 | Variability features (Time domain - 'mean hr') in peak intervals of Supine VOL |
| 114 | Variability | pi_sup_vol_4 | Variability features (Time domain - 'max hr') in peak intervals of Supine VOL |
| 115 | Variability | pi_sup_vol_5 | Variability features (Time domain - 'min hr') in peak intervals of Supine VOL |
| 116 | Variability | pi_sup_vol_6 | Variability features (Time domain - 'std hr') in peak intervals of Supine VOL |
| 117 | Variability | pi_sup_vol_7 | Variability features (Time domain - 'outlier (IQR based) ratio of peak') in peak intervals of Supine VOL |
| 118 | Variability | pi_sup_vol_8 | Variability features (Time domain - 'outlier (Z-score based) ratio of peak') in peak intervals of Supine VOL |
| 119 | Variability | pi_sup_vol_9 | Variability features (Frequency domain - 'lf') in peak intervals of Supine VOL |
| 120 | Variability | pi_sup_vol_10 | Variability features (Frequency domain - 'hf') in peak intervals of Supine VOL |
| 121 | Variability | pi_sup_vol_11 | Variability features (Frequency domain - 'lf hf ratio') in peak intervals of Supine VOL |
| 122 | Variability | pi_sup_vol_12 | Variability features (Frequency domain - 'total power') in peak intervals of Supine VOL |
| 123 | Variability | pv_tren_awp_0 | Variability features (Time domain - 'mean nni') in peak values of Trendelenburg AWP |
| 124 | Variability | pv_tren_awp_1 | Variability features (Time domain - 'rmssd') in peak values of Trendelenburg AWP |
| 125 | Variability | pv_tren_awp_2 | Variability features (Time domain - 'median nni') in peak values of Trendelenburg AWP |
| 126 | Variability | pv_tren_awp_3 | Variability features (Time domain - 'mean hr') in peak values of Trendelenburg AWP |
| 127 | Variability | pv_tren_awp_4 | Variability features (Time domain - 'max hr') in peak values of Trendelenburg AWP |
| 128 | Variability | pv_tren_awp_5 | Variability features (Time domain - 'min hr') in peak values of Trendelenburg AWP |
| 129 | Variability | pv_tren_awp_6 | Variability features (Time domain - 'std hr') in peak values of Trendelenburg AWP |
| 130 | Variability | pv_tren_awp_7 | Variability features (Time domain - 'total number of peak') in peak values of Trendelenburg AWP |
| 131 | Variability | pv_tren_awp_8 | Variability features (Time domain - 'outlier (IQR based) ratio of peak') in peak values of Trendelenburg AWP |
| 132 | Variability | pv_tren_awp_9 | Variability features (Time domain - 'outlier (Z-score based) ratio of peak') in peak values |
| 133 | Variability | pv_tren_awp_10 | Variability features (Frequency domain - 'lf') in peak values of Trendelenburg AWP |
| 134 | Variability | pv_tren_awp_11 | Variability features (Frequency domain - 'hf') in peak values of Trendelenburg AWP |
| 135 | Variability | pv_tren_awp_12 | Variability features (Frequency domain - 'lf hf ratio') in peak values of Trendelenburg AWP |
| 136 | Variability | pv_tren_awp_13 | Variability features (Frequency domain - 'total power') in peak values of Trendelenburg AWP |
| 137 | Variability | pi_tren_awp_0 | Variability features (Time domain - 'mean nni') in peak intervals of Trendelenburg AWP |
| 138 | Variability | pi_tren_awp_1 | Variability features (Time domain - 'rmssd') in peak intervals of Trendelenburg AWP |
| 139 | Variability | pi_tren_awp_2 | Variability features (Time domain - 'median nni') in peak intervals of Trendelenburg AWP |
| 140 | Variability | pi_tren_awp_3 | Variability features (Time domain - 'mean hr') in peak intervals of Trendelenburg AWP |
| 141 | Variability | pi_tren_awp_4 | Variability features (Time domain - 'max hr') in peak intervals of Trendelenburg AWP |
| 142 | Variability | pi_tren_awp_5 | Variability features (Time domain - 'min hr') in peak intervals of Trendelenburg AWP |
| 143 | Variability | pi_tren_awp_6 | Variability features (Time domain - 'std hr') in peak intervals of Trendelenburg AWP |
| 144 | Variability | pi_tren_awp_7 | Variability features (Time domain - 'outlier (IQR based) ratio of peak') in peak intervals of Trendelenburg AWP |
| 145 | Variability | pi_tren_awp_8 | Variability features (Time domain - 'outlier (Z-score based) ratio of peak') in peak intervals of Trendelenburg AWP |
| 146 | Variability | pi_tren_awp_9 | Variability features (Frequency domain - 'lf') in peak intervals of Trendelenburg AWP |
| 147 | Variability | pi_tren_awp_10 | Variability features (Frequency domain - 'hf') in peak intervals of Trendelenburg AWP |
| 148 | Variability | pi_tren_awp_11 | Variability features (Frequency domain - 'lf hf ratio') in peak intervals of Trendelenburg AWP |
| 149 | Variability | pi_tren_awp_12 | Variability features (Frequency domain - 'total power') in peak intervals of Trendelenburg AWP |
| 150 | Variability | pv_tren_vol_0 | Variability features (Time domain - 'mean nni') in peak values of Trendelenburg VOL |
| 151 | Variability | pv_tren_vol_1 | Variability features (Time domain - 'rmssd') in peak values of Trendelenburg VOL |
| 152 | Variability | pv_tren_vol_2 | Variability features (Time domain - 'median nni') in peak values of Trendelenburg VOL |
| 153 | Variability | pv_tren_vol_3 | Variability features (Time domain - 'mean hr') in peak values of Trendelenburg VOL |
| 154 | Variability | pv_tren_vol_4 | Variability features (Time domain - 'max hr') in peak values of Trendelenburg VOL |
| 155 | Variability | pv_tren_vol_5 | Variability features (Time domain - 'min hr') in peak values of Trendelenburg VOL |
| 156 | Variability | pv_tren_vol_6 | Variability features (Time domain - 'std hr') in peak values of Trendelenburg VOL |
| 157 | Variability | pv_tren_vol_7 | Variability features (Time domain - 'total number of peak') in peak values of Trendelenburg VOL |
| 158 | Variability | pv_tren_vol_8 | Variability features (Time domain - 'outlier (IQR based) ratio of peak') in peak values of Trendelenburg VOL |
| 159 | Variability | pv_tren_vol_9 | Variability features (Time domain - 'outlier (Z-score based) ratio of peak') in peak values of Trendelenburg VOL |
| 160 | Variability | pv_tren_vol_10 | Variability features (Frequency domain - 'lf') in peak values of Trendelenburg VOL |
| 161 | Variability | pv_tren_vol_11 | Variability features (Frequency domain - 'hf') in peak values of Trendelenburg VOL |
| 162 | Variability | pv_tren_vol_12 | Variability features (Frequency domain - 'lf hf ratio') in peak values of Trendelenburg VOL |
| 163 | Variability | pv_tren_vol_13 | Variability features (Frequency domain - 'total power') in peak values of Trendelenburg VOL |
| 164 | Variability | pi_tren_vol_0 | Variability features (Time domain - 'mean nni') in peak intervals of Trendelenburg VOL |
| 165 | Variability | pi_tren_vol_1 | Variability features (Time domain - 'rmssd') in peak intervals of Trendelenburg VOL |
| 166 | Variability | pi_tren_vol_2 | Variability features (Time domain - 'median nni') in peak intervals of Trendelenburg VOL |
| 167 | Variability | pi_tren_vol_3 | Variability features (Time domain - 'mean hr') in peak intervals of Trendelenburg VOL |
| 168 | Variability | pi_tren_vol_4 | Variability features (Time domain - 'max hr') in peak intervals of Trendelenburg VOL |
| 169 | Variability | pi_tren_vol_5 | Variability features (Time domain - 'min hr') in peak intervals of Trendelenburg VOL |
| 170 | Variability | pi_tren_vol_6 | Variability features (Time domain - 'std hr') in peak intervals of Trendelenburg VOL |
| 171 | Variability | pi_tren_vol_7 | Variability features (Time domain - 'outlier (IQR based) ratio of peak') in peak intervals of Trendelenburg VOL |
| 172 | Variability | pi_tren_vol_8 | Variability features (Time domain - 'outlier (Z-score based) ratio of peak') in peak intervals of Trendelenburg VOL |
| 173 | Variability | pi_tren_vol_9 | Variability features (Frequency domain - 'lf') in peak intervals of Trendelenburg VOL |
| 174 | Variability | pi_tren_vol_10 | Variability features (Frequency domain - 'hf') in peak intervals of Trendelenburg VOL |
| 175 | Variability | pi_tren_vol_11 | Variability features (Frequency domain - 'lf hf ratio') in peak intervals of Trendelenburg VOL |
| 176 | Variability | pi_tren_vol_12 | Variability features (Frequency domain - 'total power') in peak intervals of Trendelenburg VOL |

Supplementary Table 5. Hyperparameters of ML models

| Model | Hyperparameters | Values |
| --- | --- | --- |
| RF | n_estimators | 100 |
|  | max_depth | 4 |
|  | min_samples_split | 2 |
|  | min_samples_leaf | 1 |
|  | random_state | 0 |
| XGB | n_estimators | 100 |
|  | max_depth | 4 |
|  | learning_rate | 0.3 |
|  | gamma | 0 |
|  | subsample | 0.5 |
|  | min_child_weight | 1 |
|  | random_state | 0 |
| LGB | n_estimators | 100 |
|  | max_depth | 4 |
|  | learning_rate | 0.1 |
|  | min_child_weight | 0.001 |
|  | subsample | 1 |
|  | random_state | 0 |
| KNN | n_neighbors | 5 |
|  | weights | uniform |
|  | leaf_size | 30 |
| SVM | C | 1.0 |
|  | Gamma | scale |

RF: random forest, XGB: extreme gradient boosting model, LGB: light gradient boosting model, KNN: K-nearest neighbors, SVM: support vector machine.
